# The predictive value of total body PET/CT in high PD-L1 expression and immunotherapy in advanced non-small cell lung cancer patients

**DOI:** 10.3389/fonc.2025.1578419

**Published:** 2025-07-02

**Authors:** Huibin Jin, Bingxin Hu, Jie Zhang, Ye Long, Ang Xuan, Xinyu Wu, Junling Xu, Yongju Gao

**Affiliations:** ^1^ Department of Nuclear Medicine of Henan Provincial People’s Hospital and The People’s Hospital of Zhengzhou University, Zhengzhou, China; ^2^ Henan Key Laboratory of Novel Molecular Probes and Clinical Translation in Nuclear Medicine, Henan Provincial People’s Hospital, Zhengzhou, China

**Keywords:** NSCLC, SUR, PD-L1, immunotherapy, prognosis

## Abstract

**Introduction:**

Total-body positron emission tomography/computed tomography (PET/CT) using uEXPLORER scanners demonstrates superior imaging capabilities for assessing programmed death ligand 1 (PD-L1) expression heterogeneity between primary tumors (PTs) and metastatic tumors (MTs) in advanced nonsmall cell lung cancer (NSCLC).

**Methods:**

This retrospective study of 99 treatment-naïve NSCLC patients revealed that metabolic parameters (SUVmax, SUR-L, and SUR-BP) derived from biopsy-correlated PET/CT sites significantly predicted high PD-L1 expression (TC ≥ 50% or IC ≥ 10% by IHC).

**Results:**

SUR-L exhibited the highest diagnostic accuracy (AUC = 0.758, *p* < 0.001). Among 30 immunotherapytreated patients, PD-L1 positivity and SUR-BP ≥ 7.30 were associated with prolonged disease-free survival (DFS) (*p* = 0.012 and *p* = 0.035, respectively).

**Discussion:**

Our findings establish SUR-BP as a novel non-invasive biomarker for immunotherapy prognosis in NSCLC, addressing spatial heterogeneity challenges in PD-L1 assessment.

## Introduction

In recent years, immune checkpoint inhibitors (ICIs) targeting programmed death ligand 1 (PD-L1) have become an important additional cornerstone in the therapy of advanced non-small cell lung cancer (NSCLC) (1–5). Previous studies demonstrated that PD-L1 expression has two mechanisms: innate expression on tumor cells (TCs) and variable expression on tumor-infiltrating immune cells (ICs) (6, 7). Based on the European Medicines Agency (EMA) and the Food and Drug Administration (FDA), PD-L1 expression no less than 50% on TC or 10% on IC was defined as high expression, which implies that immunotherapy could be a first-line treatment for advanced NSCLC (8, 9). Therefore, it is important to get accurate PD-L1 expression levels when choosing proper treatments.

Immunohistochemistry (IHC) is widely used to detect PD-L1 expression. Generally, two kinds of specimens were tested in advanced NSCLC patients: biopsy of primary tumors (PTs) and biopsy of metastatic tumors (MTs). However, previous studies had reported the inconsistency of PD-L1 expression in different specimens (10, 11). Some studies had confirmed the temporal and spatial discordance of PD-L1 between PTs and metastases and found higher expression in PTs (12–15). However, other studies (16–18) obtained different results. The inconsistency of PD-L1 expression might be due to intramural heterogeneity in which biopsy samples could not show the panoramic view of the tumor and its microenvironment (10, 19).

β-2-^18^F-fluoro-2-deoxy-D-glucose positron emission tomography/computed tomography (^18^F-FDG PET/CT) plays a key role in tumor diagnosis, staging, re-staging, and response evaluation. Currently, the total-body PET/CT, uEXPLORER (United Imaging Healthcare, Shanghai, China) with an 194-cm-long FOV, dramatically improves image quality and the ability to detect small lesions (20, 21). Our previous studies had demonstrated that PET/CT-related parameters, including maximum standard uptake value (SUVmax) and standard uptake value ratio (SUR), had good consistency with PD-L1 expression (22–24).

Based on the above, to avoid differences between PTs and MTs in advanced NSCLC patients, we collected PD-L1 expression and PET-related parameters for both primary and metastatic sites. Thus, we conduct a retrospective study to analyze the relationship between PET/CT-related metabolic parameters obtained by the newest PET/CT machine and high PD-L1 expression in PT and MT. Furthermore, we explored the prognostic value of the expression and parameters with respect to patients’ prognosis.

## Methods

### Ethics statement

The study protocol was approved by the Institutional Review Board of Henan Provincial People’s Hospital. Our ethics committee waived the need for informed consent from the study participants. All methods were performed in accordance with the relevant guidelines and regulations in our analysis.

### Patients

In this study, 99 patients with primary NSCLC from June 2020 to March 2022 in our hospital were enrolled. The screening criteria were as follows: (1) first diagnosis of NSCLC without other systemic diseases or treatments; (2) integrity of pathological data; (3) total body PET/CT images before biopsy; and (4) first treated at our hospital during the study period. Clinicopathological data included age, gender, maximum diameter, smoking history, histological subtype, the source of histologic samples, metastatic sites, and treatments. The study protocol was approved by the institutional review board, and the need for written informed consent was waived.

### ^18^F-FDG PET/CT

All patients fasted no less than 6 h and serum glucose levels were no more than 10 mmol/L before intravenous injection of ^18^F-FDG with a dosing regimen (3.7 MBq/kg). All patients rested approximately 60 min after injection and then underwent PET/CT imaging. All images were acquired on total-body PET/CT (uEXPLORER, United Imaging Healthcare, Shanghai, China). A low-dose CT scan (tube current, 10 mA; voltage, 100 kV; rotation time, 0.5 s; pitch, 1.0125; collimation, 80 × 0.5 mm) was conducted first for anatomical localization and reconstructed in a 512 × 512 matrix for attenuation correlation. Then, PET imaging was performed with 5-min acquisition.

### Image analysis

All images were analyzed by two experienced nuclear medicine physicians with one of them having at least 10 years of experience. Region of interest (ROI) was drawn at lung primary lesions and metastatic lesions on PET/CT images and SUVmax was calculated based on body weight. Meanwhile, mean standard uptake value (SUVmean) of liver and blood pool were collected. A 30-mm-diameter ROI was placed at the normal right hepatic lobe to avoid intrahepatic lesions. A 10-mm-diameter ROI was placed at the middle of the descending aorta to avoid partial volume effects. SUR values were defined as the ratios of lung lesions/metastatic lesions SUVmax to liver and blood pool SUVmean (SUR-L and SUR-BP, respectively).

### Immunohistochemical staining

All tissues were fixed with 10% formalin after no less than 6 h and embedded in paraffin, and then hematoxylin–eosin (HE) staining and IHC were conducted. All samples were analyzed on an automated stainer with 22C3 (PD-L1 test kits, DAKO/Agilent, USA) (25). At least two pathologists evaluated the slides to determine the scores of PD-L1-positive cells on TC and/or IC. According to clinical trials (1, 2), PD-L1 high expression was defined as positive scores on TC of no less than 50% or IC no less than 10%.

### Response assessment

All patients’ treatment response information was retrospectively acquired from electronic medical records and patients’ imaging and testing results. Disease-free survival (DFS) was used to conduct a response assessment over a median follow-up of 5.53 (0.17–34.07) months in our patients. DFS was defined as the time from diagnosis until disease progression (positive) or the last visit in our hospital where the patient was alive without recurrence (negative). All treatment protocols were the first line. As for the immunotherapy group, those patients were undergoing immunotherapy only or in combination with chemotherapy. As for the “others” group, those patients were undergoing chemotherapy, targeted therapy, or radiotherapy. The response to therapy was assessed on 4 August 2023.

### Statistical analysis

Statistically significant differences were analyzed using chi-square test or Fisher’s exact test for categorical variables and Mann–Whitney U test for continuous variables. Receiver operating characteristic (ROC) curve analyses were used to test the continuous variables and discriminate negative and positive PD-L1 expression; sensitivity (Se) and specificity (Sp) were collected to choose the optimal cutoff value, and the 95% confidence intervals (CIs) were calculated. The log-rank test with Kaplan–Meier analysis was used to make survival analysis. All statistical analyses were carried out with SPSS, version 23.0 (SPSS Inc., Chicago, IL, USA). Statistical significance was defined as *p* < 0.05.

## Results

### Patients’ clinical characteristics

Clinical characteristics are shown in **Table 1**. There were 99 patients enrolled in the research with 20 squamous cell carcinoma (SCC) and 79 adenocarcinoma (ADC). Among these 99 patients, the biopsy of 62 patients was from the PTs and that of 37 patients was from the MTs. A total of 37 patients (37.4%) expressed PD-L1 positively with 23 (37.1%) in biopsy of PTs and 14 (37.8%) in MTs. As for MTs, 27 (73%) were from lymph nodes, 5 (13.5%) from bone, 2 (5.4%) from brain, 1 (2.7%) from pleura, and 2 (5.4%) from pleural fluid.

**Table 1:** Characteristics of PD-L1 expression.

Characteristics	PD-L1				DFS			
	Negative (62)	Positive (37)	χ^2^	P	Negative (52)	Positive (47)	χ^2^	P
Age (year)			0.081	**0.775**			0.358	**0.550**
<63	25	16			23	18		
≥63	37	21			29	29		
Gender			0.126	**0.722**			0.356	**0.551**
Male	38	24			34	28		
Female	24	13			18	19		
Maximum Diameter (mm)			1.475	**0.225**			0.055	**0.814**
<30	26	11			20	17		
≥30	36	26			32	30		
Smoking history			0.001	**0.980**			0.518	**0.472**
Smoker	30	18			27	21		
Non-smoker	32	19			25	26		
Histologic subtype			3.327	**0.068**			0.562	**0.454**
ADC	53	26			40	39		
SCC	9	11			12	8		
Biopsy			0.005	**0.941**			0.033	**0.857**
Primary tumors	39	23			33	29		
Metastatic tumors	23	14			19	18		
Metastatic tumors			4.184	**0.382**			1.211	**0.876**
Lymph node	16	11			13	14		
Bone	3	2			3	2		
Brain	2	0			1	1		
Pleura	0	1			1	0		
Pleural fluid	2	0			1	1		
Treatments			2.932	**0.087**			2.708	**0.100**
Immunotherapy	15	15			12	18		
Others	47	22			40	29		

PD-L1, programmed death ligand 1; DFS, disease-free survival; NSCLC, non-small cell lung cancer; ADC, adenocarcinoma; SCC, squamous cell carcinoma; immunotherapy, immunotherapy only or combination with chemotherapy; others, chemotherapy, targeted therapy or radiotherapy.Bold value, p value.

Among all the patients, a total of 30 patients received immunotherapy and 69 patients received other first-line treatments. A total of 47 patients (47.4%) had disease progression, with 18 (60%) patients receiving immunotherapy and 29 (42%) receiving the other treatments. Of the patients, 58 (58.6%) were younger than 63 years, 62 (62.6%) were male patients, 62 (62.6%) had a diameter of no less than 30 mm, and 48 (48.5%) were smokers. None of these characteristics were correlated with PD-L1 expression or DFS.

### ^18^F-FDG PET parameters

**Figure 1:**
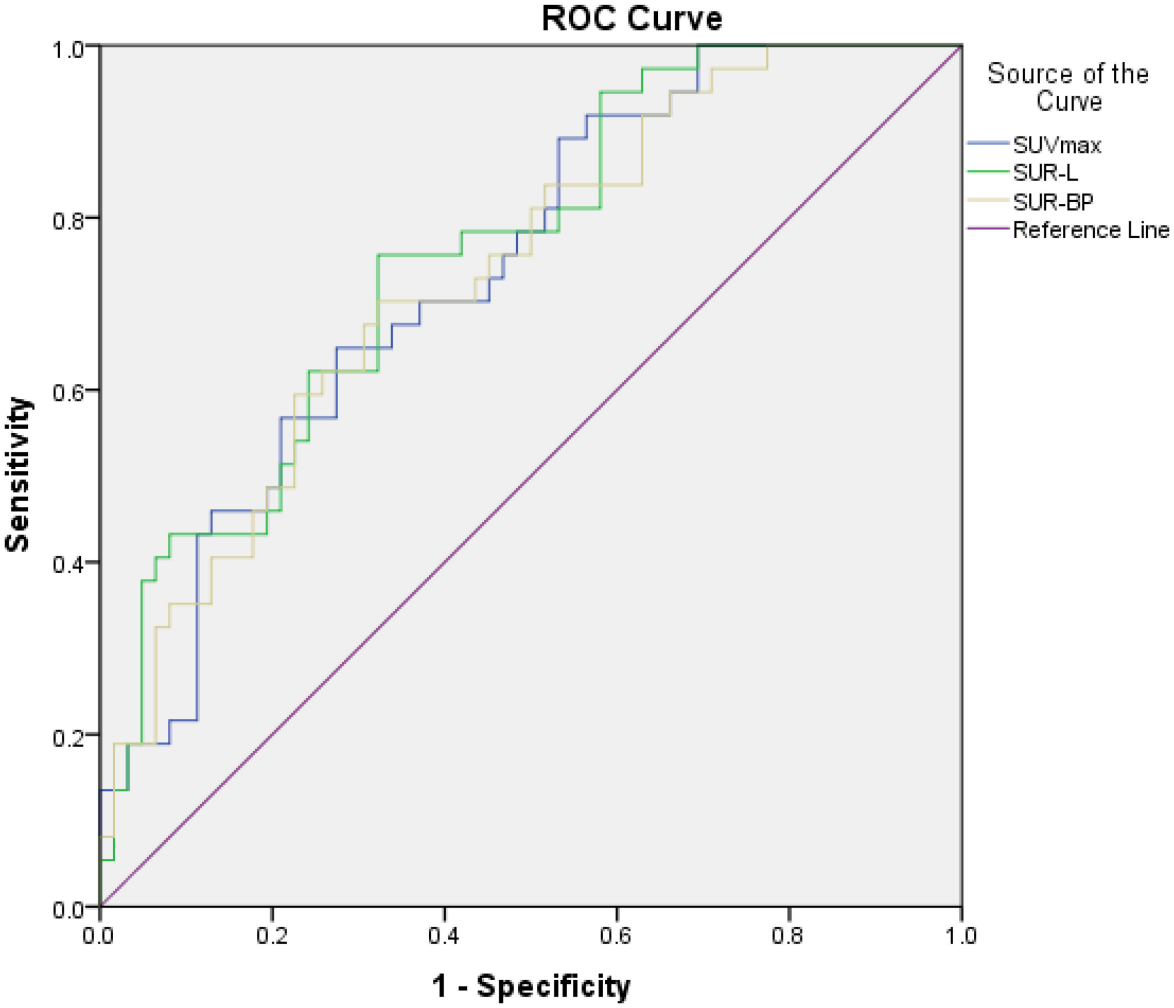
The relationship between PET parameters and high PD-L1 expression. The area under curve (AUC), cutoff value, sensitivity, and specificity of SUVmax were 0.739, 13.29, 64.9%, and 72.6%, respectively; those of SUR-L were 0.758, 4.97, 75.7%, and 67.7%, respectively; and those of SUR-BP were 0.735, 7.30, 70.3%, and 67.7%, respectively.

The correlation between PET-related parameters and PD-L1 expression is shown in **Table 2** and **Figure 1**. PET/CT parameters were acquired from both primary and metastatic lesions, ensuring spatial concordance with biopsy sites.

**Table 2:** The relationship between PET parameters and PD-L1 expression.

Parameters	Numerical value (mean±SD)			
	PD-L1(-)(62)	PD-L1(+)(37)	U	P
SUVmax	10.29±5.50	16.29±9.06	599	0.000*
SUR-L	4.67±2.91	7.78±4.17	555	0.000*
SUR-BP	6.87±4.36	11.51±6.96	608	0.000*

*: P<0.05; SD, standard deviation; U, Mann-Whitney U test; SUVmax, the maximum of standard uptake value; SUR-L, the ratio of lung lesion SUVmax to liver SUVmean; SUR-BP, the ratio of lung lesion SUVmax to blood pool SUVmean.

In our patients, those metabolic parameters were higher in positive than in negative, including SUVmax (16.29 ± 9.06 vs. 10.29 ± 5.50, *p* < 0.001), SUR-L (7.78 ± 4.17 vs. 4.67 ± 2.91, *p* < 0.001), and SUR-BP (11.51 ± 6.96 vs. 6.87 ± 4.36, *p* = 0.001). The best cutoff value of SUVmax determined by ROC was 13.29, and the area under curve (AUC) was 0.739 (95% CI: 0.642–0.836, *p* < 0.001) with a sensitivity (Se) of 64.9% (95% CI: 47.5%–79.8%) and a specificity (Sp) of 72.6% (95% CI: 59.8%–83.2%). The best cutoff value of SUR-L determined by ROC was 4.97, and the AUC was 0.758 (95% CI: 0.663–0.853, *p* < 0.001) with a Se of 75.7%(58.8%–88.2%) and a Sp of 67.7% (54.7%–79.1%). The best cutoff value of SUR-BP determined by ROC was 7.30, and the AUC was 0.735 (95% CI: 0.636–0.834, *p* = 0.000) with a Se of 70.3% (53.0%–84.1%) and a Sp of 67.7% (54.7%-79.1%). It could be seen that SUR-L, which had the largest AUC, demonstrated moderate diagnostic accuracy.

**Figure 2:**
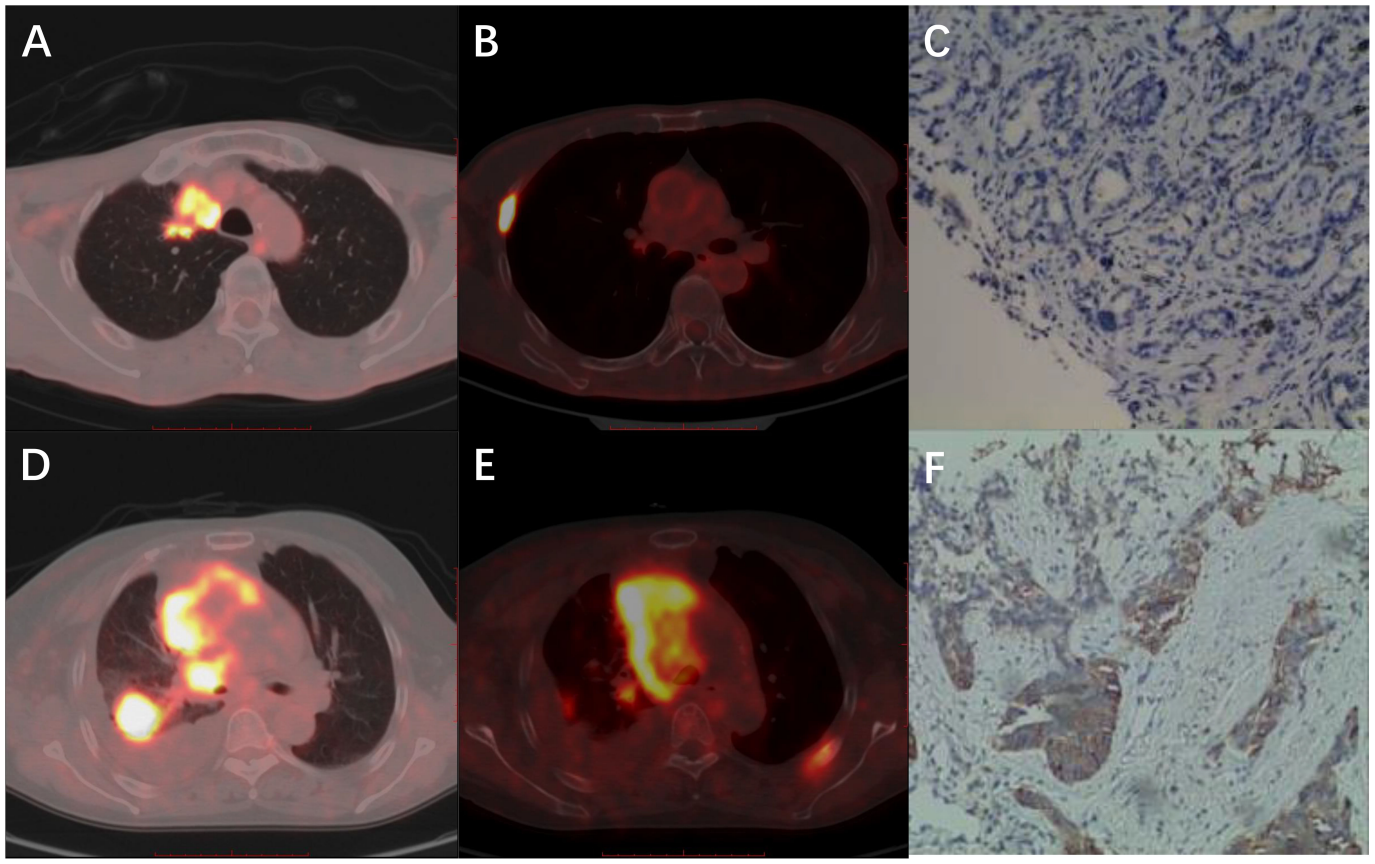
Representative images of PET/CT and IHC. **(A-C)** A 68-year-old man with ADC. The biopsy site was from rib metastasis and PD-L1 expression was negative. The SUVmax, SUR-L, and SUR-BP of primary tumors were 15.72, 6.89, and 9.14 and of rib metastasis were 19.92, 8.74, and 11.58. **(D-F)** A 66-year-old woman with SCC. The biopsy site was from lung primary tumors and PD-L1 expression was positive. The SUVmax, SUR-L, and SUR-BP of primary tumors were 14.76, 5.35, and 10.32, and those of lung primary tumors were 5.23, 1.89, and 3.66, respectively.

The PET/CT and IHC images are shown in **Figure 2**. The first patient (A–C) was a 68-year-old man with ADC. The biopsy site was from a rib metastasis and PD-L1 expression was negative. The SUVmax, SUR-L, and SUR-BP of PTs were 15.72, 6.89, and 9.14, and those of rib metastasis were 19.92, 8.74, and 11.58, respectively. The second patient (D–F) was a 66-year-old woman with SCC. The biopsy site was from lung PTs and PD-L1 expression was positive. The SUVmax, SUR-L, and SUR-BP of PTs were 14.76, 5.35, and 10.32 and those of lung PTs were 5.23, 1.89, and 3.66, respectively.

### Prognostic value of PD-L1 expression and PET parameters

**Figure 3:**
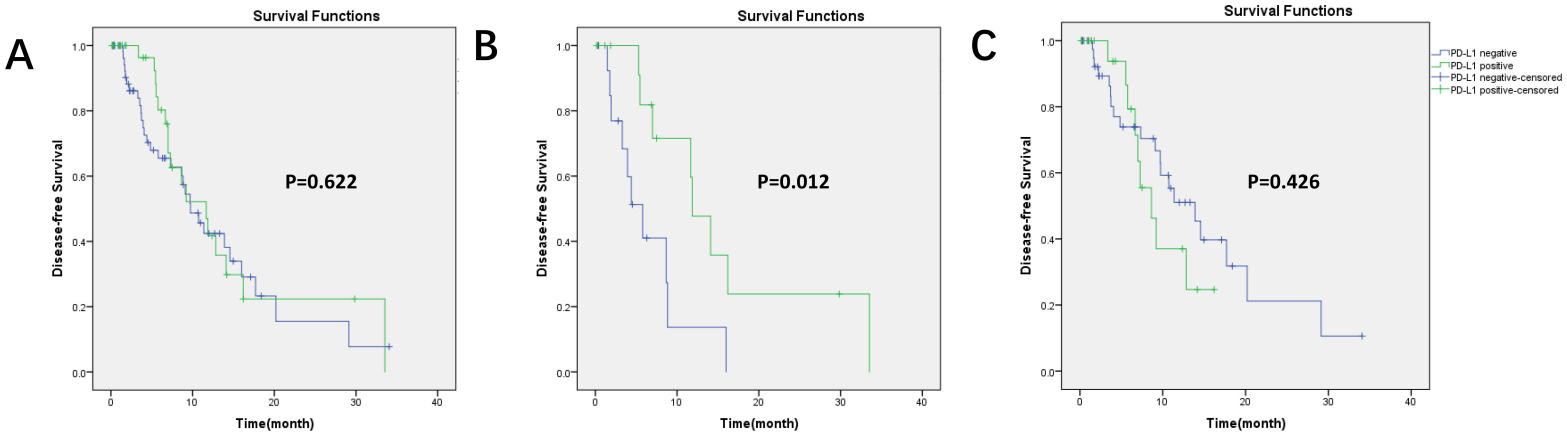
Kaplan–Meier survival curves for DFS based on PD-L1 expression. **(A)** In all patients (99 patients), *p* = 0.622; **(B)** in immunotherapy (30 patients), *p* = 0.012, PD-L1 positivity had longer DFS; **(C)** in other treatments (69 patients), *p* = 0.426.

**Figure 4:**
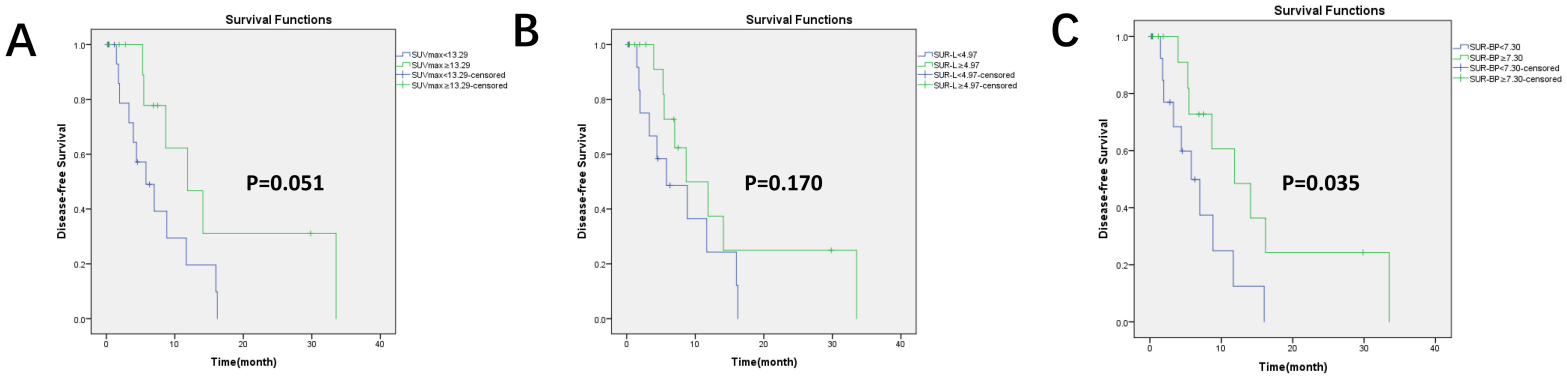
Kaplan–Meier survival curves for DFS based on the optimal value of PET/CT parameters in the immunotherapy group. There were 30 patients who received immunotherapy. **(A)** Based on the optimal cutoff value of SUVmax = 13.29, *p* = 0.051; **(B)** based on the optimal cutoff value of SUR-L = 4.97, *p* = 0.170; **(C)** based on the optimal cutoff value of SUR-BP = 7.30, *p* = 0.035; SUR-BP no less than 7.30 had longer DFS.

Kaplan–Meier survival curves for DFS based on PD-L1 expression and PET parameters are shown in **Figures 3**, **4**. In our group, there were 30 patients undergoing immunotherapy only or combination with chemotherapy and 69 patients undergoing other treatments, including chemotherapy, targeted therapy, or radiotherapy. During our follow-up process, a total of 47 people had disease progression. PD-L1 expression was not related to DFS in all patients (*p* = 0.622) and it was the same with the other treatments (*p* = 0.426). However, PD-L1 positivity had a significantly longer DFS (*p* = 0.012) than PD-L1 negativity in patients who received immunotherapy.

By applying a cutoff value of PD-L1-positive expression in SUVmax (13.29), SUR-L (4.97), and SUR-BP (7.30), we found that DFS was longer in SUR-BP ≥ 7.30 for those who received immunotherapy only or combination with chemotherapy. There was no significant difference in the other groups. SUR-BP ≥ 7.30 was the only correlating factor for DFS in our analysis.

## Discussion

ICIs targeting PD-1/PD-L1 have prolonged the survival time in patients with advanced NSCLC, and PD-L1 is the only biomarker used for screening patients for ICIs. Previous studies had pointed out that PD-L1 expression was inconsistent in PT and MT. Based on these studies, PD-L1 expression data were collected from PTs and MTs of patients with advanced lung cancer, as well as the relevant PET parameters from PET/CT machines corresponding to the biopsy sites. To our knowledge, this represents the first investigation of the predictive value of PET/CT parameters for immunotherapy outcomes in patients with advanced NSCLC.

Some studies have investigated PD-L1 expression in NSCLC between PTs and MTs and found significant discrepancy between the two (10–13, 16–18). Generally, the expression was higher in PTs than that in MTs. Previous studies (22, 23, 26–28) had confirmed that PET/CT parameters were related to PD-L1 expression in lung cancer. The PET/CT parameters included SUVmax, SUVmean, SUVpeak, SUR, metabolic tumor volume (MTV), and total lesion glycolysis (TLG). These studies included patients with stage I–IV disease, but the studies mentioned did not specifically distinguish the source of the specimens between the primary and metastatic lesions.

For better patient management, in our study, for patients whose biopsy sites were from MT, we collected PET/CT-related parameters from those biopsy sites to ensure sites’ consistency between the PD-L1 expression and PET/CT parameters. In this regard, we found that SUVmax, SUR-L, and SUR-BP were associated with PD-L1 expression but not with clinical factors. In PET/CT data, SUR-L had the biggest AUC, which was 0.758. Therefore, our research indicated that the stability of SUR was superior to SUVmax, which was consistent with previous studies (29, 30).

In our study, all patients whose biopsy sites were from MT also had PET parameters from the corresponding MT. To compare with other studies, we also analyzed PET/CT parameters in those 99 patients’ PTs with PD-L1 expression (regardless whether the biopsy sites were from PT or MT); the AUC of PT-SUVmax, PT-SUR-L, and PT-SUR-BP was 0.675, 0.696, and 0.683, respectively (not shown). These results were in line with previous studies (22–24, 31). However, it was clear that the PET/CT parameters corresponding to the biopsy sites had greater reference value, which indicated that PET/CT parameters had good correlation with PD-L1 expression. In our group, PET/CT parameters in primary lesions were higher than those in metastatic lesions (not shown). Kaira et al. pointed out that PD-L1 expression was linked to hypoxia-inducible factor alpha (HIF-α) and glucose transporter 1 (GLU1) (32). Hence, our metabolic values might support the idea that PD-L1 expression was lower in MT (12, 13).

Some studies concluded that both PD-L1 expression and PET/CT parameters were predictive factors to survival outcomes (32–36). However, others did not support the conclusion (26, 37–39). In our group, when we did not separate immunotherapy from the other treatments, the survival analysis showed no significant correlation between PD-L1 expression and DFS, as well as PET/CT parameters. Such different results might be related to several factors, such as treatment protocols, tumor types, patients’ selection, and stage.

Kudura et al. (40) pointed out that PD-L1 expression and PET/CT parameters were very strong long-term outcome predictors of patients treated with immunotherapy, while no significant outcome predictors could be found for the cohort with no immunotherapy. To avoid treatments bias, we performed analysis on immunotherapy and other treatments separately. Our results were in line with them. In patients who received immunotherapy as first line, we found that PD-L1 positivity and having a SUR-BP no less than 7.30 had favorable clinical outcomes, which was consistent with previous research (41). What was more important, we were the first to report that SUR-BP was associated with PD-L1 expression and immunotherapy outcomes.

In the immunotherapy subgroup, PET/CT parameters were acquired from both primary and metastatic lesions, ensuring spatial concordance with biopsy sites. In addition, we also analyzed the relationship between the primary lesion PET/CT parameters and prognosis. The *p*-value of PT-SUVmax, PT-SUR-L, and PT-SUR-BP was 0.384, 0.047, and 0.224 by Kaplan–Meier analysis, respectively. The results indicated that PT-SUR-L had a higher reference value for the prognosis of immunotherapy. In our follow-up, positive DFS was due to the progression of the metastatic lesions. This might explain the difference between the two sets of results.

There were some limitations in our study: First, the absence of paired PD-L1 measurements in PT–MT pairs limits our ability to characterize intrapatient heterogeneity. Future studies incorporating multi-site synchronous biopsies are warranted. Second, we only collected DFS without overall survival (OS) and progression-free survival (PFS). If we included these two factors, we could provide more comprehensive prognostic information. Third, as for PET/CT parameters, we lacked MTV and TLG, among other parameters (42, 43). They also had some influence on PD-L1 expression and clinical outcomes. More parameters should be included in future studies in order to obtain more accurate prognostic information. Fourth, our study was single-center, retrospective, and based on a Chinese cohort; thus, the results might have some limitations. Multi-center, prospective, and multi-population studies might yield more authoritative results.

## Conclusion

SUVmax, SUR-L, and SUR-BP values were consistent with PD-L1 expression. PD-L1 expression and SUR-BP were related to DFS. In immunotherapy, PET/CT parameters could provide relevant reference values for PD-L1 expression and treatment prognosis, especially SUR-BP.

## Data Availability

The raw data supporting the conclusions of this article will be made available by the authors, without undue reservation.
